# CausalR: extracting mechanistic sense from genome scale data

**DOI:** 10.1093/bioinformatics/btx425

**Published:** 2017-06-29

**Authors:** Glyn Bradley, Steven J Barrett

**Affiliations:** Target Sciences, GSK, Medicines Research Centre, Stevenage, UK

## Abstract

**Summary:**

Utilization of causal interaction data enables mechanistic rather than descriptive interpretation of genome-scale data. Here we present CausalR, the first open source causal network analysis platform. Implemented functions enable regulator prediction and network reconstruction, with network and annotation files created for visualization in Cytoscape. False positives are limited using the introduced Sequential Causal Analysis of Networks approach.

**Availability and implementation:**

CausalR is implemented in R, parallelized, and is available from Bioconductor

**Supplementary information:**

Supplementary data are available at *Bioinformatics* online.

## 1 Introduction

With the continuing increase in the generation of genome-wide expression datasets, the limitations of commonly used interpretation methods, such as Gene Ontology classification, Gene Set Enrichment Analysis (Subramanian *et al.*, 2005) and pathway mapping, have become increasingly apparent. They are useful for classifying endpoints extracted from a new experiment but are of limited use at uncovering the mechanisms that led to the observed changes.

Causal reasoning (causal network analysis) can be used to predict the root cause of observed effects. It requires a causal graph, in the form of a signed, directed interaction network, describing the system under study. Observed endpoints serve as a starting point for the analysis. A reasoning algorithm is then used to track back through the causal graph to find points of convergence that maximally and accurately explain the differential regulatory pattern seen across the endpoints. In a biological analysis these points are likely to be key upstream regulators of the observed endpoints, and so for example in a drug discovery environment may represent the best targets for reversing the observed endpoints.

Here we present CausalR, a biologically focused causal reasoning implementation coded in the popular statistical language R, and made available from the Bioconductor project (Gentleman *et al.*, 2004). CausalR builds upon existing methodology (Chindelevitch *et al.*, 2012a, b) and provides an enhanced, open source alternative to commercial software (Kramer *et al.*, 2014).

## 2 Input data

### 2.1 Causal networks

CausalR runs on networks in the simple interaction format (.sif), where interactions take the form:

**Table:** 

ProteinA	Activates	ProteinB
ProteinC	Inhibits	ProteinD

Although the majority of causal information currently resides in the commercial domain, publicly available repositories do exist (e.g. Perfetto *et al.*, 2015), and large public databases such as IntAct (Orchard *et al.*, 2014) have recently started to curate into causal form.

### 2.2 Experimental data

CausalR experimental input data take the form of differential expression (or activity) levels between case and control. This could be the output of any ‘omics experiment, but in reality is usually differentially expressed gene signatures resulting from transcriptomics analysis. CausalR analysis utilizes the direction, but not magnitude, of change and so for input, gene signatures are summarized to the form:

**Table:** 

GeneX 1
GeneY 0
GeneZ −1

where ‘1’ denotes up-regulation, ‘0’ denotes unchanged and ‘−1’ denotes down-regulation. High-quality gene expression data, generated from human lung fibroblast cells treated with various cytokines and suitable for the generation of test input gene signatures, are available on the Gene Expression Omnibus (Edgar *et al*., 2002) at accession number GSE60880.

## 3 Examples and usage

### 3.1 Example workflow and data

**Fig. 1.**
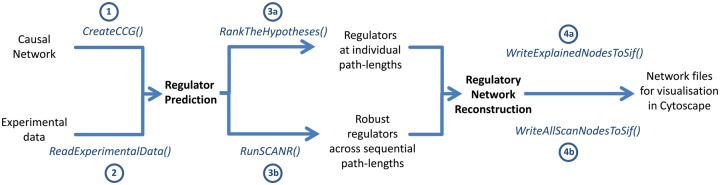
An example CausalR workflow, to predict regulators of input experimental data and generate regulatory networks

As an example, we will demonstrate the CausalR functionality depicted in Figure 1, to predict regulators of input experimental transcriptional data and reconstruct regulatory networks explaining the regulation pattern of genes seen in the transcriptional data. As test input experimental data we will use the IL1B time-course treatment from GSE60880, which yielded IL1B response signatures at 1, 2 and 8 h. The CausalR ready signatures are provided in the Supplementary Material.

We will use an input causal network extracted from publicly available interaction data manually curated from the literature, focused on immune regulation (see Catlett *et al.*, 2013). Full details and the network itself are also provided in the Supplementary Material, along with example R scripts.

### 3.1 Input processing

The first step in CausalR analysis is the creation of a computational causal graph (CCG) from the input substrate causal network, using the *CreateCCG()* function (labelled 1 in Fig. 1) The experimental data are then read in and mapped against the CCG using the *ReadExperimentalData()* function (2 in Fig. 1). Here CausalR will give a warning telling the user how many of the input genes are not represented in the input causal network. It is of course preferable to have as many of the input genes represented in the causal network as possible, however due to the sparsity of causal information currently in the public domain it is likely unrealistic to expect close to 100%.

### 3.3 Regulator prediction

Upstream regulators of the input experimental data can then be predicted using the *RankTheHypotheses*() and/or *runSCANR*() function(s). *RankTheHypotheses*() (3a in Fig. 1) can be used to predict regulators at individual path lengths, and is minimally parameterized with the name of the CCG and experimental data (both now stored in the R workspace), and the user-supplied delta parameter, which specifies the maximum number of edges (path-length) to be traversed within the CCG from a (signed) regulator hypothesis to an outcome signal node for a predictive path to be scored for that combination. Each node in the causal network is ranked for its fit with the input experimental data. In the case of the example IL1B data the true upstream regulator is known, and IL1B does indeed rank highly in the tabulated results (see Supplementary Table S1) output from *RankTheHypotheses*() for all three of the time course signatures. This gives confidence there is enough relevant information in the CCG to run network reconstruction.

In experimental situations where the true regulators are not known and CausalR is being employed to predict them, it is useful to limit false positives. The *runSCANR*() function (3b in Fig. 1) executes the Sequential Causal Analysis of Networks (SCAN) methodology, novel to CausalR. This approach repeats the *RankTheHypotheses*() function multiple times by iterating over a user-supplied sequence of increasing delta (path length) values. It then scans across those sets of results to uncover common regulator hypotheses appearing within the top ranked genes, as defined by the user-supplied topNumGenes parameter.

The theory behind this approach is that hypotheses predicted to control genes across multiple path lengths are more likely to be true regulators than those only predicted to control genes at a single path-length. SCAN thus provides the ability to uncover potentially more robust, biologically plausible regulator hypotheses.

### 3.4 Regulatory network reconstruction

A Cytoscape ready .sif file comprising the regulatory network for an individual CausalR predicted regulator and associated annotation file are constructed via the *WriteExplainedNodesToSIF*() function (4a in Fig. 1). This function takes as inputs the CCG, the input experimental data, the regulator hypothesis and the number of path lengths to be searched. If the user has applied the SCAN methodology, networks for all resulting SCAN nodes can be automatically generated using the *WriteAllScanNodesToSIF*() function (4b in Fig. 1).

**Fig. 2.**
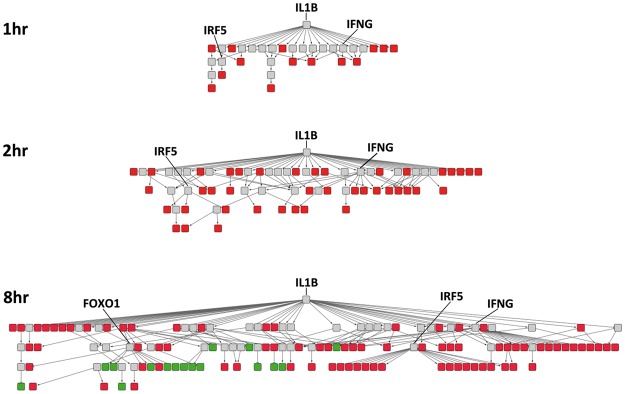
CausalR reconstructed IL1B response networks. CausalR analysis was carried out on gene signatures from a time course of IL1B treatment of human lung fibroblasts. Visualization of the resulting networks in Cytoscape shows how the signalling response develops. Coloured nodes represent genes upregulated (red) or downregulated (green) in the input experimental data, and so being explained by these signalling networks. Grey nodes represent genes not changed in the experimental input but predicted to be part of the signalling cascade

The networks produced by *WriteExplainedNodesToSIF*() for the time-course IL1B gene signatures are given in Figure 2. Such visualizations are an intuitive way of describing the increased complexity of the signalling response as the time course of IL1B treatment progresses, with stimulatory effects over 1 and 2 h and feedback inhibition loops becoming apparent only after 8 h. The top hubs in the 8 h response network, IFNG, FOXO1 and IRF5 are highlighted. All three are known to play a key role in IL1B signalling (Masters *et al.*, 2010; Su *et al.*, 2009; Duffau *et al.*, 2015), giving confidence in the CausalR reconstructed networks.

## 4 Conclusion

CausalR provides causal reasoning (causal network analysis) methods for the Bioconductor project. We hope it will both help researchers extract mechanistic sense from genome-scale data, and serve to stimulate interest in the development of these and associated techniques in the academic community. Increased curation of causal interaction data in the public domain is an important component for further developments in this area.

## Supplementary Material

Supplementary Data

Supplementary Test Data
