# Supplementary material for: CausalR: extracting mechanistic sense from genome scale data
Source: Bioinformatics. 2017 Jun 29;33(22):3670–2. doi: 10.1093/bioinformatics/btx425 (PMC5870775; doi:10.1093/bioinformatics/btx425)
Supplement: Supplementary Data [file causalr_supplementaryinformation_btx425.docx]

**Package Design and Implementation**

**Dependencies**

CausalR was developed for the R statistical computing environment (Hornik, 2015), and is released as a package (Bradley et al., 2016) on Bioconductor (Gentleman, et al., 2004). It depends on R version >=3.0, and the R edition of the igraph network analysis software (Csardi & Nepusz, 2006), available at the igraph website (<http://igraph.org/r/>) and mirrors of the Comprehensive R Archive Network (<https://cran.r-project.org/>). The Cytoscape network analysis software (Shannon et al., 2003) is required for visualisation of some outputs. The package builds are available for Windows, Linux and Mac.

**Design**

The code is written following software design principles to provide functions that have a single purpose, minimising the amount of interdependency (i.e. provides low coupling) whilst encapsulating highly cohesive functionality into modules. CausalR consists of:

- 69 functions, 22 exposed to the enduser
- 19 igraph R functions incorporating C code, called by 17 functions not exposed to the end-user.

- 6 core modules:

FileReader
- reads and processes the input data files into R objects, representing the signed directed network and the subset of experimental data with overlapping nodes. The network is represented as a computational causal graph, where each hypothesis node is featured as separately signed nodes for up- and -down regulation.

Predictions
- This is where the relevant information is extracted from the network in preparation for doing the scoring and significance calculations, i.e. it finds and creates a matrix of all possible predictions containing causal relations between each (signed) hypothesis (a network node) and nodes from a matching re-constructed experimental data matrix.

Scoring
- calculates, for each root signed hypothesis, the number of correct, ambiguous and incorrect paths, of up to path-length (input parameter) delta , to all it's downstream connected (node) components of the input experimental signal present within the network. Calculates the overall hypothesis score (=#correct - #incorrect) and returns all this infomation to the Predictions module.

Significance
- Computes p-values given the hypothesis score and distribution table via quartic [exact] or cubic [approximate] methods (Chindelevitch etal., 2012) . Calculates enrichment p-values via Fisher Exact test (fisher.test function in base R) for a given hypothesis by comparing predicted with observed changes from experimental data.

RankingHypothesis
- constructs the final results table, calling functions from each of the Predictions, Scoring and Significance modules. Sends individual hypothesis vector information from the Predictions module matrix to the scoring and significance calculator modules to generate rows of values which it uses to construct and then rank the overall scores matrix.

Utilities
- comprise the runSCANR functionality for finding regulator hypotheses common across a range of path lengths, and means for constructing their regulatory networks.

**Optimisation and Parallelisation**

The two 'workhorse' top-level end-user functions, RankTheHypotheses and runSCANR, are optionally supported by parallelisation using the integrated doParallel and parallel packages found in the base R installation (Weston and Calaway, 2015). The number of available compute nodes to use can be specified by the user or automatically detected by the software. Operation is seamless across the --supported operating systems.

The Predictions module function RankTheHypotheses has various options to allow speed-up of calculations:
- due to the properties of the CCG, predictions and scores for half of the CCG (i.e. down-regulated hypothesis nodes) can be obtained faster through symmetry.
- the exact (quartic) or faster approximate (cubic 1b) algorithm (see Chindelevitch et al., 2012) can be selected for p-values calculations.
- runScanR allows switching on/off p-values calculation for hypotheses below a supplied score threshold parameter, correctPredictionsThreshold , whose default setting is inf (infinity, i.e. switched-off).

**Testing**

All functions and modules have been comprehensively tested prior to package construction and over 80% unit testing coverage has been exposed to all OS builds available at Bioconductor.

**Documentation**

The package is supported with a comprehensive application vignette (CausalR Developers Team, 2016) and detailed, R style online support for all functions in the package. The main package description and basic usage information covering the most important end-user functions is accessed by typing ?CausalR in the R console window, whilst ??CausalR returns further, more detailed information on underlying (called) functions (and their default settings) as well as a link to the vignette.

**Inputs and Basic Functionality**

The core functionality CausalR provides is to compute how well the predictions from each hypothesis (a node from the input network with a direction of regulation) match the provided experimental data; this is expressed as a score. The significance of these scores is calculated using two different techniques adapted from Chindelevitch et al., 2012b. Finally the nodes are ranked by their scores to produce a list of ordered likely causes for what is seen in the experimental results, which can be subsequently filtered by applying significance thresholding. Regulatory networks for highly ranking, high confidence hypotheses can then be extracted from the network for further visualisation.

CausalR requires at least two inputs to the file reader module functions:

(1) A network data file in Cytoscape.sif format, containing a list of stimulatory or inhibitory interactions specified as follows:

GENENAME1 Activates GENENAME2
GENENAME3 Inhibits GENENAME4

This comprises the global substrate causal network information used by CausalR to construct a CCG to reason through, to generate regulator hypotheses and their downstream networks.

For the illustrative example used within this application note, the network was constructed from interactions extracted from the BEL Large Corpus v1.4, (accessed from <http://resource.belframework.org/belframework/1.0/knowledge/large_corpus.bel>).

The network is available in file causalNetwork.sif. Users should note the license conditions of the original source.

(2) tab-delimited text file containing the experimental (differential gene signal) data:

GENENAME5 1
GENENAME7 0
GENENAME1 -1

GENENAME4 1
GENENAME9 0

Gene names with 1's and -1's represent up- and down-regulated genes respectively, as defined by having differential expression above (or below) an upper (or lower) fold-change cut-off. Gene names with corresponding zeros distinguish those with fold change below the absolute value of the cuts. Note that whilst inclusion of genes failing the fold change cut-off greatly expands the signal and increases CausalR run-times, they are needed for full assessment of ambiguous outcomes and accurate p-value calculations. Excluding these however markedly decreases runtimes and does not affect the individual hypothesis scores used for ranking. Table S1 summarises how this information is used in scoring hypotheses and table S2 shows an example results.

**Table S1.** **Hypothesis Scoring:** The following table summarises how the individual paths from a hypothesis node to a signal node is scored. In the absence of a unique shortest path to any given outcome, more than one path will be evaluated, with the possibility disagreement between predicted outcome node regulations. These are counted as ‘ambiguous’ in the results table and do not contribute to scoring.

| **Score = Σ( *Sum*√_h_ – *Sum*X_h_ )** | | **PREDICTED HYPOTHESIS GENE REGULATION** | | |  |
| --- | --- | --- | --- | --- | --- |
|  | **LABEL** | **↑** | **↓** | **↔ / ≠** | **>δ** |
| **MEASURED (Experiment)** | **↑** | **√** | **X** | **Ã** |  |
|  | **↓** | **X** | **√** | **Ã** |  |
|  | **↔** | **Ã** | **Ã** | **Ã** |  |
| **UNMEASURED** |  |  |  |  |  |

**Ã denotes ambiguous (counts of these are ignored at δ =1)**

**↔ denotes unchanged, which can only be predicted as up or down regulation.**

**≠ denotes disagreement between predictions from multiple paths from a single hypothesis node to a signal node.**

| NodeName | NodeID | Regulation | Score | Correct | Incorrect | Ambiguous | p-value | Enrichment p-value |
| --- | --- | --- | --- | --- | --- | --- | --- | --- |
| TNF | 1351 | 1 | 35 | 43 | 8 | 1 | 0.00E+00 | 2.55E-09 |
| IRF5 | 653 | 1 | 27 | 29 | 2 | 0 | 0.00E+00 | 1.32E-09 |
| MEOX2 | 800 | -1 | 21 | 27 | 6 | 0 | 0.00E+00 | 1.14E-10 |
| IL1B | 616 | 1 | 17 | 20 | 3 | 1 | 3.48E-06 | 6.41E-05 |
| IRF7 | 654 | 1 | 15 | 18 | 3 | 0 | 4.73E-05 | 1.06E-04 |
| IL18 | 612 | 1 | 13 | 14 | 1 | 0 | 2.11E-06 | 2.06E-04 |
| IFNG | 584 | 1 | 13 | 18 | 5 | 1 | 4.43E-04 | 6.41E-05 |
| NGF | 876 | 1 | 11 | 19 | 8 | 0 | 3.13E-02 | 1.27E-03 |
| IFNA1 | 580 | 1 | 10 | 10 | 0 | 0 | 5.14E-06 | 9.48E-04 |
| --- |  |  |  |  |  |  |  |  |
| IFNA1 | 580 | -1 | -10 | 0 | 10 | 0 | 1.00E+00 | 9.48E-04 |
| IL1A | 615 | -1 | -10 | 1 | 11 | 0 | 1.00E+00 | 3.56E-05 |
| NGF | 876 | -1 | -11 | 8 | 19 | 0 | 9.83E-01 | 1.27E-03 |
| IFNG | 584 | -1 | -13 | 5 | 18 | 1 | 1.00E+00 | 6.41E-05 |
| IL18 | 612 | -1 | -13 | 1 | 14 | 0 | 1.00E+00 | 2.06E-04 |
| IRF7 | 654 | -1 | -15 | 3 | 18 | 0 | 1.00E+00 | 1.06E-04 |
| IL1B | 616 | -1 | -17 | 3 | 20 | 1 | 1.00E+00 | 6.41E-05 |
| MEOX2 | 800 | 1 | -21 | 6 | 27 | 0 | 1.00E+00 | 1.14E-10 |
| IRF5 | 653 | -1 | -27 | 2 | 29 | 0 | 1.00E+00 | 1.32E-09 |
| TNF | 1351 | -1 | -35 | 8 | 43 | 1 | 1.00E+00 | 2.55E-09 |

**Table S2. - Example Results Table with ranked regulator hypotheses for the IL1B signal**  Only the head and foot of the results table is shown to display the scoring symmetry. Reflecting all +/- signed hypotheses evaluated, the results table output by the RankTheHypotheses function will always contain twice as many entries as there are nodes in the input network.

The IL1B signatures used in the illustrative example in this application note were generated as follows: Human lung fibroblast cells were incubated with IL1B, 1, 2, and 8 hours. Total RNA was prepared and global transcriptomic profiling carried out by standard methods on Affymetrix U113A Genechips. The data has been deposited in the GEO (GSE60880). RMA normalisation and quality control was carried out in affy and ArrayQualityMetrics packages the R/Bioconductor statistical environment. Differentially expressed gene signatures were obtained by ANOVA analysis, in the ArrayStudio software (Omicsoft), using 2 fold change and 0.05 FDR cut-offs. The fold change values were summarised to the direction of change (ie 1 or -1) to run CausalR. Those genes unchanged in the experiment were set to 0 to facilitate p-value calculations. CausalR ready signatures are given in supplementary data files IL1B_1hr_signature.txt, IL1B_2hr_signature.txt and IL1B_8hr_signature.txt

**References**

Bradley G., Barrett S., Wille D., Bonde B., Woollard P., Mistry C., Riley D. and Pipe M. CausalR: Causal Reasoning Methods. R package version 1.4.3. 2016. <https://www.bioconductor.org/packages/release/bioc/html/CausalR.html>

CausalR Developers Team. Overview of Causal Reasoning with CausalR : Hypothesis Scoring and P-value Calculation. 2016. <https://www.bioconductor.org/packages/release/bioc/vignettes/CausalR/inst/doc/CausalR.pdf>

Chindelevitch, L., Loh, P., Enayetallah A., Berger B. and Ziemek D. Assessing statistical significance in causal graphs (Methodology article). BMC Bioinformatics 2012;13:35- 48. .

Csardi G. and Nepusz T. The igraph software package for complex network research, InterJournal, Complex Systems 1695. 2006. <http://igraph.org>

Fischer, R. A. Statistical Methods for Research Workers. Oliver & Boyd. 1970

Gentleman, R.C., et al. Bioconductor: open software development for computational biology and bioinformatics. Genome Biol 2004;5(10):R80. <https://www.bioconductor.org>

Hornik, Kurt (November 26, 2015). "R FAQ". The Comprehensive R Archive Network. 2.13 What is the R Foundation? <https://cran.r-project.org/doc/FAQ/R-FAQ.html>

Shannon,P. , Markiel,A., Ozier,O., Baliga,N., Wang,J., Ramage,D., Amin,N., Schwikowski,B., and Ideker, T. Cytoscape: A Software Environment for Integrated Models of Biomolecular Interaction Networks. Genome Res. 2003;13(11): 2498–2504. <http://www.cytoscape.org/>

Weston, S. and Calaway, R. Getting Started with doParallel and foreach. 2015. <https://cran.r-project.org/web/packages/doParallel/vignettes/gettingstartedParallel.pdf>
